# Enhanced magnetic resonance imaging manifestations of paediatric intervertebral disc calcification combined with ossification of the posterior longitudinal ligament: case report and literature review

**DOI:** 10.1186/s12887-022-03461-5

**Published:** 2022-07-08

**Authors:** Cancan Chang, Juan Zhu, Hongyi Li, Qing Yang

**Affiliations:** 1 Department of Medical Imaging, Bozhou Hospital of Traditional Chinese Medicine, Bozhou, 236800 Anhui China; 2 Clinical Medicine Standardisation Training Trainees, Department of Medical Imaging, Anqing Hospital Affiliated to Anhui Medical University, Anqing, 246000 Anhui China; 3 Department of Medical Imaging, Anqing Hospital Affiliated to Anhui Medical University, Anqing, 246000 Anhui China; 4 Department of Radiology, The People’s Hospital of Liaoning Province, Shenyang, 110016 China; 5 Department of Medical Imaging, Anqing Hospital Affiliated to Anhui Medical University, Renmin road, Anqing, 246000 Anhui China

**Keywords:** Intervertebral disc calcification, Ossification of the posterior longitudinal ligament, Paediatric, Enhanced magnetic resonance imaging, case report

## Abstract

**Background:**

Since the first description of paediatric intervertebral disc calcification (IDC) by Báron in 1924, only approximately 400 cases have been reported in the literature. Paediatric IDC combined with ossification of the posterior longitudinal ligament (OPLL) is an even rarer condition, with only 8 cases described in detail to date. In this paper, we present a review of the disease characteristics described in the relevant English language literature and discuss the possible mechanisms of lesion enhancement in contrast-enhanced magnetic resonance imaging (MRI).

**Case presentation:**

In May 2020, a 6-year-old Han nationality girl presented with the chief complaint of neck pain that had lasted for a week. She did not report a history of trauma or a past illness. On admission, there was no personal and family history, congenital diseases, or non-specific infections such as tuberculosis, among others. Further physical examination revealed that the movement of her cervical spine was limited. Computed tomography (CT) and MRI revealed ossification of the intervertebral discs and posterior longitudinal ligament (PLL) at the C4/5 levels and an absence of obvious spinal cord compression. When contrast-enhanced MRI was performed, significant enhancement was observed in the intervertebral discs and PLL at the C4/5 level. We adopted a non-interventional approach and performed an imaging re-examination 8 months later. Both the plain and contrast-enhanced MRI scans indicated swelling in the C4/5 intervertebral discs and disappearance of the previously observed enhancement in the nucleus pulposus (NP) and PLL at the corresponding levels; CT examination revealed that the ossified lesions had been completely resorbed.

**Conclusion:**

Obvious lesion enhancement in contrast-enhanced MRI is an extremely rare manifestation of paediatric IDC combined with OPLL. However, the exact mechanisms of this phenomenon remain unclear. We surmise that it may be caused by a series of biophysical changes related to vertebral endplate injury and repair, but further research will be required for in-depth investigation.

## Background

Paediatric (0–18 years) or juvenile intervertebral disc calcification (IDC) is a rare, typically self-limiting disease of an unknown origin. Since the first report of pain in the thoracic vertebrae, pyrexia, and scoliosis in a 12-year-old boy by Báron in 1924, only approximately 400 cases in the 0–20-year age group have been reported in the literature [1–5]. Ossification of the posterior longitudinal ligament (OPLL) mainly affects individuals of Asian descent aged 50–70 years and is a disease of unknown aetiology characterised by the replacement of ligamentous tissue by new ectopic bone [6]. Paediatric IDC is generally considered to be self-limiting with good prognosis, whereas OPLL in adults usually worsens gradually and requires surgical treatment upon the occurrence of myelopathy or radiculopathy. Under certain circumstances, the onset of IDC and OPLL may occur concurrently. Such events are extremely rare, with only 9 cases described in the literature [7]. The initial x-ray and computed tomography (CT) images of patients with IDC revealed the presence of high-density masses in the intervertebral space, and T2-weighted magnetic resonance imaging (MRI) showed reduced signal intensity in the involved intervertebral discs. Contrast-enhanced MRI scans performed on a handful of IDC patients showed mild or no obvious lesion enhancement. Among the patients with IDC combined with OPLL reported in the literature (excluding the present case study), only one had undergone contrast-enhanced MRI, which revealed mild lesion enhancement. By contrast, the patient of this case study manifested obvious enhancement of the calcified masses and ossified posterior longitudinal ligament (PLL), which differs considerably from the previously reported presentations. As the aetiology of this syndrome remains unclear, a few possible mechanisms are proposed in the ensuing discussion.

## Case presentation

**Fig. 1:**
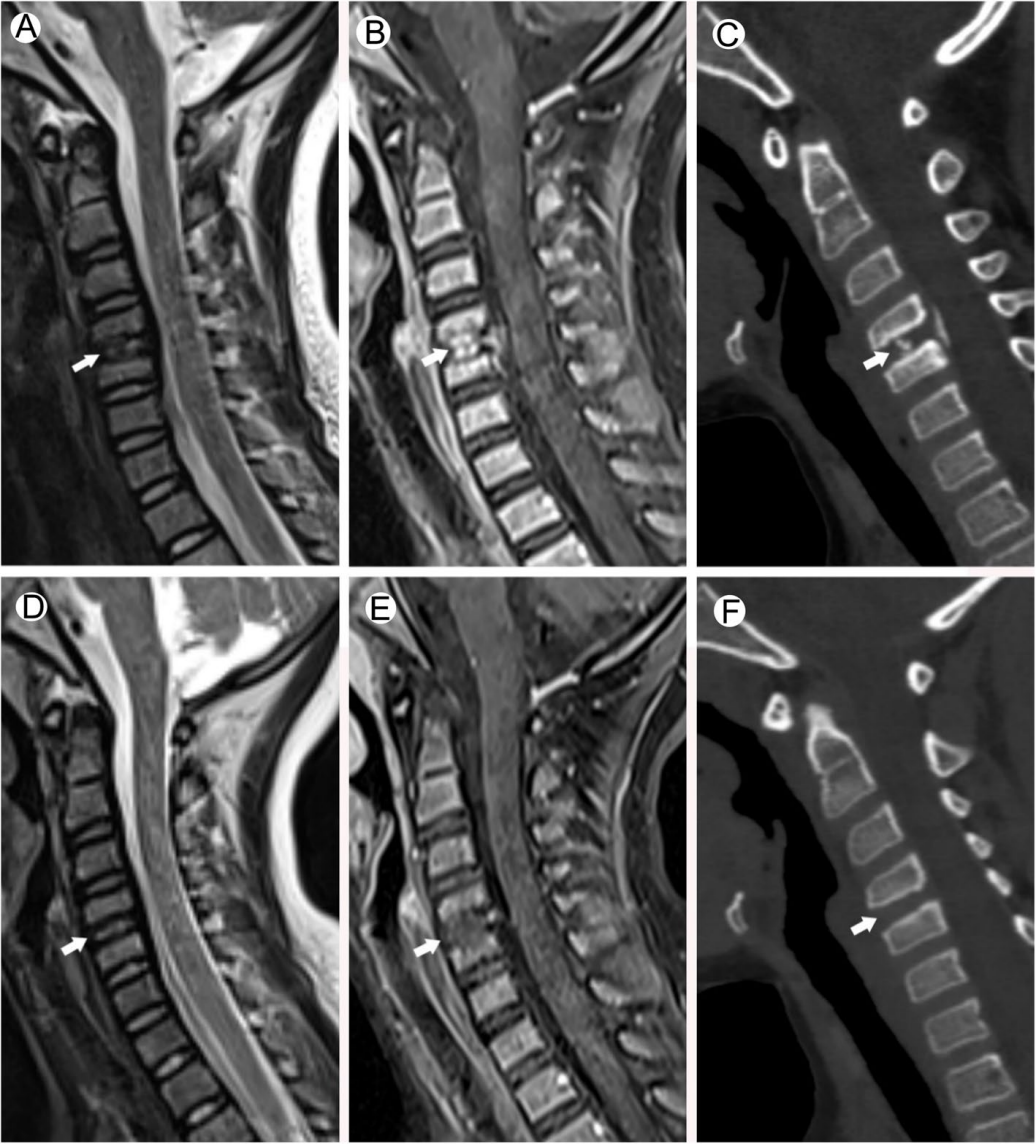
Radiological imaging at admission (**A**, **B**, **C**). The sagittal T2WI (**A**) of the child at admission shows a partially reduced signal in the intervertebral disc. The sagittal T1-FS enhanced examination (**B**) shows patchy enhancement in the nucleus pulposus from the C4/C5 intervertebral disc and the corresponding posterior longitudinal ligament area. Cervical CT sagittal view (multi-planar reformation, MPR image) (**C**) shows calcification of the C4/C5 intervertebral disc, ossification of the posterior longitudinal ligament at the C4/C5 level, shortened longitudinal axes of the vertebral bodies of C4 and C5, and a heterotrophic calcific deposit at the endplate of the opposite edges of the vertebral bodies. After 8 months of follow-up, the sagittal images of the same patient (**D**, **E**, **F**) show that the signal from the C4/C5 disc is restored, the enhancement of the original disc and posterior longitudinal ligament disappears, and the disc calcification and posterior longitudinal ligament ossification also completely disappears

In May 2020, a 6-year-old Han nationality girl presented with the chief complaint of neck pain that had lasted for a week. She didn’t take any medical treatment, but 3 days after the initial onset, her symptoms did not improve. She did not report a history of trauma, infection, or past illness. On admission, there was no personal and family history, congenital diseases, or non-specific infections such as tuberculosis, among others. Physical examination revealed that the movement of her cervical spine was limited. No torticollis nor palpable mass was found. Further neurological examination didn’t reveal apparently motor dysfunction or myodynamia deficit. A plain MRI scan revealed a localised reduction of the T2-weighted signal and soft tissue swelling in the PLL (Fig. 1A) at the C4/5 intervertebral discs. Contrast-enhanced MRI (T1 FS sequence) after intravenous injection of a gadolinium-based contrast agent indicated obvious enhancement of masses in the nucleus pulposus (NP) and PLL at the C4/5 level (Fig. 1B). A preliminary diagnosis of inflammatory lesions of intervertebral discs with concomitant abscess formation in the PLL was considered. However, results of laboratory examinations, including inflammatory markers, complete blood count, calcium level, and phosphorus level, were within the normal range, which was inconsistent with this diagnosis. To achieve a more comprehensive examination, CT scans were performed and the results indicated the presence of calcification in the C4/5 NP and localised PLL ossification posterior to the C4/5 vertebral bodies. We also observed axial height reduction in the C4 and C5 vertebrae and endplate sclerosis in the opposite edges of the C4 and C5 vertebrae (Fig. 1C). Based on the CT examination results and relevant literature, a final diagnosis of paediatric IDC combined with OPLL was made.

Considering that the disease was benign and self-limited, and the patient’s symptoms were not very severe, we adopted a non-interventional approach. At 8-month-follow-up visit, the patient’s symptoms greatly improved and we performed an imaging re-examination. Both the plain and contrast-enhanced MRI scans indicated swelling in the C4/5 intervertebral discs and disappearance of the previously observed enhancement in the NP and PLL at the corresponding levels (Fig. 1D and E). CT examination showed the disappearance of both the C4/5 NP calcification and localised PLL ossification posterior to the C4/5 vertebral bodies (Fig. 1F). The patient got a satisfactory recovery.

## Discussion and conclusion

IDC is a rare disease that affects the intervertebral discs and the structures of surrounding vertebrae, muscles, and ligaments. It usually results in local pain or sensorimotor disorders. The potential aetiologies and pathophysiological mechanisms of the disease remain unclear, but IDC is typically self-limiting, with clinical symptoms disappearing spontaneously within several weeks or months [8]. IDC has an extremely low incidence, with only approximately 400 cases reported in the literature since it was first described in 1924. OPLL is characterised by the replacement of ligamentous tissue by new ectopic bone. Its aetiology is currently unknown and is mainly studied by researchers in Japan [9, 10]. The prevalence of OPLL is 0.1–1.7% in Europe and the United States, 0.4–3% in Asia (excluding Japan), and 1.9–4.3% in Japan [11]. OPLL mainly affects individuals aged 50–70 years, with the mean age of onset being 51.2 years for men and 48.9 years for women. The disease can be classified into four subtypes based on morphology, namely localised (localised to the intervertebral disc space), segmental (ossification behind each vertebral body), continuous (ossification that spans several vertebral bodies), and mixed (a mixture of both segmental and continuous types) [12]. IDC combined with OPLL is extremely rare in children, with only 8 cases reported in the current literature (Table 1).

**Table 1:** Summary of published cases of children with IDC combined with OPLL

Reference	Reported Year	Age/Sex	Locations	Pre-existing trauma	Clinical presentation	WBC (/mm3)/CRP (mg/L)/ESR (mm/h)	Magnetic resonance contrast enhancement	Treatment	Outcome/follow-up
Zhu et al. [13].	2019	8 years old/F	IDC at C5/6, OPLL at C5/6	No	NP&ND&BP	5950/13.4/67	NO	No-intervention	Fully recovery/6 months
Du et al. [4].	2012	8 years old/F	IDC at C6/7, OPLL at C6/7	Yes	NP& ND	5860/16.5/55	NO	Conservative treatment	Fully recovery/2 years
Du et al. [4].	2012	6 years old/M	IDC at C2/3, C3/4, OPLL at C3/4	No	NP	6170/11.8/69	NO	Conservative treatment	Mild IDC remain/18 months
Fu et al. [14].	2011	11 years old/M	IDC at T6/7, T7/8, OPLL at T6/7	No	BP	Normal	No	Conservative treatment	Aggravated IDC/3 months
Wang et al. [7].	2016	11 years old/F	IDC at C5/6, OPLL at C5/6, C6	No	NP	Normal	NO	Conservative treatment	Mild IDC remain/6 months
Li et al. [15].	2016	7 years old/F	IDC at C3/4, C4/5, OPLL at C3/4	No	NP	–	Yes	Conservative treatment	Fully recovery/1 year
Mizukawa et al. [16].	2017	6 years old/F	IDC at C4/5, OPLL at C4/5	No	NP	8600/15/−	No	Conservative treatment	Fully recovery/6 months
Odell et al. [17].	2016	9 years old/M	IDC at C2/3, OPLL at C2/3	Yes	NP & stiffness； torticollis	–	No	Conservative treatment	Fully recovery/3 months
Current patient	2020	6 years old/F	IDC at C4/5, OPLL at C4/5	No	NP	Normal	Yes	No-intervention	Fully recovery/8 months

– Unknown

*IDC* intervertebral disc calcification, *OPLL* ossification of posterior longitudinal ligament, *WBC* white blood cells, *CRP* C-reactive protein, *ESR* erythrocyte sedimentation rate, *NP* neck pain, *ND* neurological deficit, *BP* back pain

Combined analysis of the relevant literature (8 cases + the current case study) revealed that the median age of patients with IDC combined with OPLL was 8 years (6–11 years) and the ratio of boys to girls was 3:6. The condition was not significantly correlated with trauma, with only 2 of the 9 patients having a definite history of trauma. The vast majority of patients exhibited non-specific clinical symptoms and were only diagnosed by chance through imaging examinations [18]. White blood cell count was within the normal range in all cases, whereas a slight increase in C-reactive protein was detected in a number of patients. IDC combined with OPLL may involve any of the vertebral levels. We found that the most commonly affected vertebral level was the C4/5 level (*n* = 5, 22.7%), followed by the C3/4 level (*n* = 4, 18.2%), C5/6 level (*n* = 4, 18.2%), C2/3 level (*n* = 3, 13.6%), C6/7 level (*n* = 2, 9.1%), T7/8 level (*n* = 1, 4.5%), and C6 level (*n* = 1, 4.5%). Lesions mainly involved the cervical spine; a possible explanation for this phenomenon offered by Zhu et al. [13] is that the cervical spine is subjected to the greatest amount of activity within the entire vertebral column. Therefore, excessive movements may result in greater load and injury in the intervertebral discs and accessory structures, and the subsequent inflammatory reaction will cause further damage to the NP and surrounding structures, ultimately inducing the onset of IDC and OPLL. We agree with this hypothesis, as the most important function of the intervertebral discs is mechanical—to transfer loads, dissipate energy, and allow movement in the vertebral column [19]. Excessive loads may upset the existing balance and cause endplate injury, thereby affecting the physiological state of the intervertebral discs.

We summarized the imaging manifestations of IDC combined with OPLL in children in previous relevant research and our case (Table 2). A review of the 8 cases in the literature revealed that contrast-enhanced MRI of the cervical spine was only performed in 2 cases. In the case report by Li et al. [15], mild enhancement was observed in the ossified masses after gadolinium injection, but abnormal enhancements were not found in the adjacent intervertebral discs. Therefore, an in-depth discussion of the findings was not offered. In another case reported by Dönmez et al. [20], no obvious enhancement was found in the T1-weighted images of the calcified intervertebral discs. In the present study, a plain MRI scan revealed intervertebral disc swelling, localised reduction of the T2-weighted signal, and soft tissue swelling in the PLL (Fig. 1A) at the C4/5 intervertebral discs (Fig. 1A). Contrast-enhanced MRI (T1 FS sequence) after intravenous injection of a gadolinium-based contrast agent indicated obvious enhancement of masses in the nucleus pulposus (NP) and PLL at the C4/5 level (Fig. 1B). These findings are markedly different from those reported in the two case studies described earlier and indicate that calcification may be considerably enhanced in MRI, contrary to the conventional belief that it merely presents as non-enhanced or mildly enhanced masses. The possible reasons for this difference are discussed below.

**Table 2:** Summary of the imaging manifestations of IDC combined with OPLL in children

Reference	Locations	X-ray/CT /MRI				MRI	
		Physical curvature	Decreased vertebral height	Expansion of the intervertebral disk space	Spinal canal encroachment	Hypointensity in the affected disk space on T2WI	Contrast-enhanced MRI
Zhu et al. [13].	IDC at C5/6, OPLL at C5/6	Straighten	Yes	Yes	Yes	Yes	Not scanned
Du et al. [4].	IDC at C6/7, OPLL at C6/7	Straighten	Yes	Yes	Yes	Yes	Not scanned
Du et al. [4].	IDC at C2/3, C3/4, OPLL at C3/4	Slightly Straighten	Yes	Yes	Yes	Yes	Not scanned
Fu et al. [14].	IDC at T6/7, T7/8, OPLL at T6/7	Straighten	Yes	Yes	Yes	Not scanned	Not scanned
Wang et al. [7].	IDC at C5/6, OPLL at C5/6, C6	Straighten	Yes	Yes	Yes	Yes	Not scanned
Li et al. [15].	IDC at C3/4, C4/5, OPLL at C3/4	Straighten	Yes	Yes	Yes	Yes	Mildly lesion enhancement
Mizukawa et al. [16].	IDC at C4/5, OPLL at C4/5	Slightly Straighten	Yes	Yes	Yes	Not scanned	Not scanned
Odell et al. [17].	IDC at C2/3, OPLL at C2/3	Slightly Straighten	Yes	Yes	Yes	Not scanned	Not scanned
Current patient	IDC at C4/5, OPLL at C4/5	Straighten	Yes	Yes	Yes	Yes	Obvious lesion enhancement

*IDC* Intervertebral disc calcification, *MRI* Magnetic resonance imaging, *OPLL* Ossification of the posterior longitudinal ligament, *T2WI* T2 weighted image

**Fig. 2:**
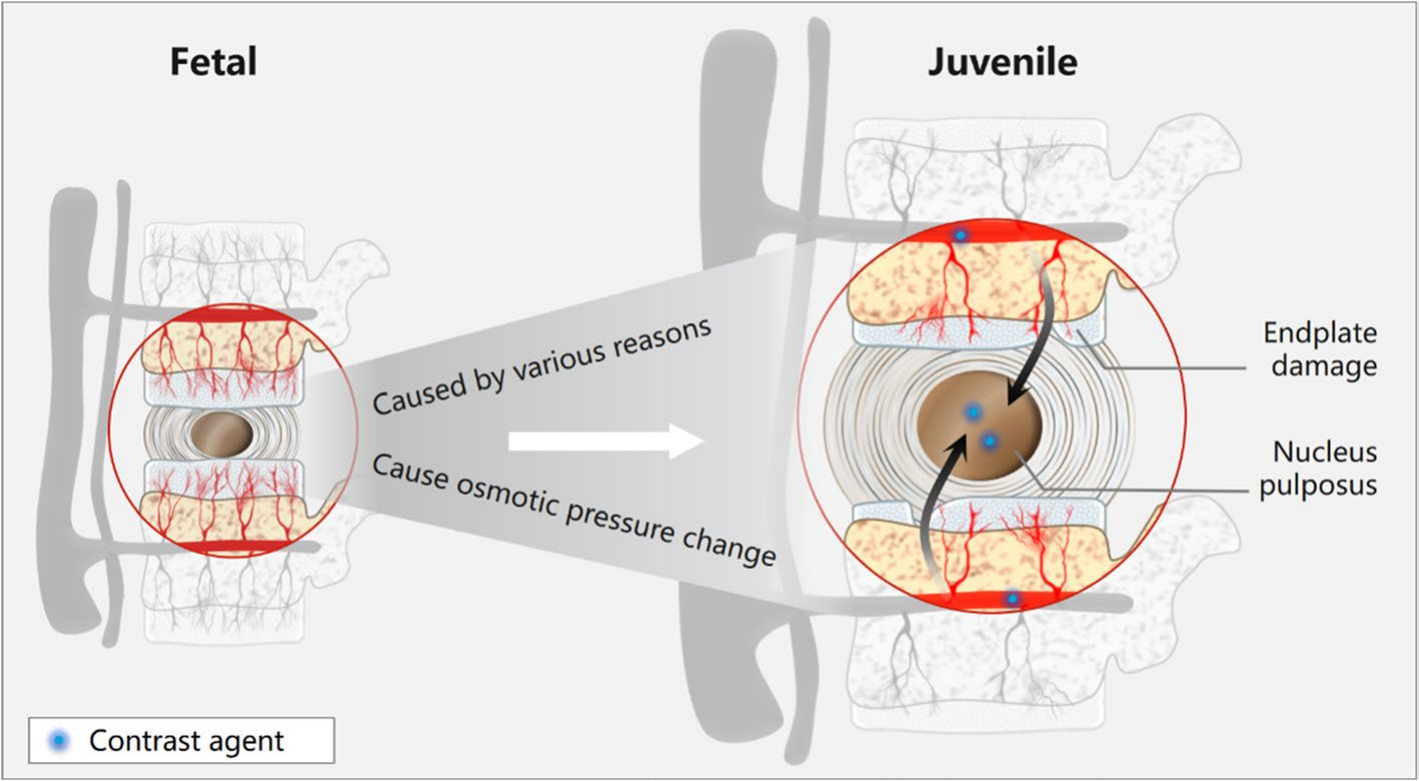
This figure shows the schematic diagram of the mechanisms underlying structural changes and calcification of intervertebral discs from foetal age to teenage as identified by contrast-enhanced MRI. From foetal age to teenage, the thickness of endplates and the density of nutrient vessels decrease gradually. In this process, various factors (inflammation, trauma, genes, etc.) cause injury or fracture of the vertebral endplates, resulting in delayed disappearance of endplates, leakage of contrast agent into the nucleus pulposus, and annihilation of calcification of the intervertebral discs, thus leading to significant enhancement of the calcified mass

Nerlich et al. [21] stated that in the intervertebral discs of foetal to infantile age, blood vessels perforate the cartilaginous endplate and extend into the inner and outer annulus fibrosus, but not into the nucleus pulposus. By contrast, blood vessels are completely absent in the intervertebral discs of adolescents and adults with the exception of areas near the ligament attachment points in the outer anulus fibrosus [19]. In the absence of a blood supply, diffusion is the only source of nutrition to the intervertebral discs, and alteration of diffusion is considered to be the final common pathway for disc degeneration [22]. Rajasekaran et al. [23] utilised contrast-enhanced MRI for the analysis of diffusion in human lumbar discs and found that the endplate is the main structure that controls the process of diffusion. Results indicated that peak enhancement was attained first in the vertebral body and then sequentially in the subchondral bone, endplate, peripheral NP, and central NP. In a normal disc with an intact endplate, the diffusion process is characterised by a march-like pattern with a delay in peak enhancement in the endplate (termed “endplate delay”). In other studies that performed postoperative analyses of the pathological features of IDC [24], the vast majority of pathological findings showed the absence of neovascularisation within the calcified masses. Therefore, a possible explanation for the obvious enhancement of calcified masses in the present study may be endplate injury or fracture, which can result in the disappearance of endplate delay, leakage of contrast agent into the NP, and obscuring of calcification by the contrast agent (Fig. 2). Our imaging results also indicated obvious enhancement of swelling in the NP of the calcified intervertebral discs, which was closely associated with the contiguous vertebral endplate region, and the presence of abnormal calcium deposits in the contiguous vertebral endplate and subchondral bone. Such manifestations may be indicative of degenerative endplate calcification, which will lead to endplate injury and affect nutrient metabolism in the NP of intervertebral discs, ultimately resulting in NP calcification. In other words, alterations in the vertebral bodies may have preceded those of the intervertebral discs. This is consistent with the view held by Swischuk et al. [25] that calcific discitis does not merely indicate IDC but also includes disc swelling, which may have occurred before calcification and involved the vertebral bodies.

When all cases of IDC combined with OPLL in the literature were retrospectively analysed, it was found that OPLL occurred in the ligament connecting two vertebral bodies at the site of IDC in almost all cases, including the present case. Such OPLL belongs to the localised subtype, and there is a need to investigate the cause of this phenomenon. Chen et al. [26] held the view that OPLL plaques may be the result of extension from enthesis sites in the vertebral body. Another study [27] reported that the ossified mass is always in direct contact with the cortical bone and that OPLL plaques are contiguous with the ligamentous enthesis to the vertebral body and to the deep layer of the PLL. In the present case study, our findings seem to suggest the presence of a connection between the linear enhancement at the posterior edge of the C5 vertebral body and the enhanced ossified masses. This may have been the site of origin of OPLL, which agrees with the conjecture proposed in previous research. However, there is a lack of histological evidence and samples of enhanced OPLL to support this hypothesis. Obvious enhancement of the OPLL ossification region may possibly be explained by the early histological changes associated with OPLL. These include hyperplasia of fibroblast-like and chondroblast-like cells, infiltration of small vessels, and endochondral ossification [12], with small vessel infiltration being a possible cause of enhancement.

IDC combined with OPLL is extremely rare in children with an unknown origin. MRI revealed obvious lesion enhancement in contrast-enhanced MRI, an extremely rare manifestation of paediatric IDC combined with OPLL. However, we must be aware of this possibility to avoid unnecessary excessive treatment or even surgery for the patient. The exact mechanisms of the enhancement remain unclear. We surmise that it may be caused by a series of biophysical changes related to vertebral endplate injury and repair, but further research will be required for in-depth investigation.

## Data Availability

All the data supporting our findings is contained within the manuscript.

## Abbreviations

CRP: C-reactive protein
CT: Computed tomography
ESR: Erythrocyte sedimentation rate
IDC: Intervertebral disc calcification
MRI: Magnetic resonance imaging
OPLL: Ossification of the posterior longitudinal ligament
WBC: White blood cells
NP: Nucleus pulposus
PLL: Posterior longitudinal ligament

## References

[1] Lam SK, Niedzwecki CM, Daniels B, Mayer RR, Vakharia MM, Jea A (2016). Acute spinal cord injury associated with multilevel pediatric idiopathic intervertebral disc calcification: case report. J Neurosurg Pediatr.

[2] Dushnicky MJ, Okura H, Shroff M, Laxer RM, Kulkarni AV (2019). Pediatric idiopathic intervertebral disc calcification: single-center series and review of the literature. J Pediatr.

[3] Sieroń D, Gruszczyńska K, Machnikowska-Sokołowska M, Olczak Z, Knap D, Baron J (2013). Intervertebral disc calcification in children: case description and review of relevant literature. Pol J Radiol.

[4] Du JJ, Meng H, Cao YJ, Li FQ, Luo ZJ (2012). Calcification of the intervertebral disc and posterior longitudinal ligament in children. J Spinal Disord Tech.

[5] Chu J, Wang T, Pei S, Yin Z (2015). Surgical treatment for idiopathic intervertebral disc calcification in a child: case report and review of the literature. Childs Nerv Syst.

[6] Matsunaga S, Sakou T (2012). Ossification of the posterior longitudinal ligament of the cervical spine: etiology and natural history. Spine (Phila Pa 1976).

[7] Wang G, Kang Y, Chen F, Wang B (2016). Cervical intervertebral disc calcification combined with ossification of posterior longitudinal ligament in an-11-year old girl: case report and review of literature. Childs Nerv Syst.

[8] Tsutsumi S, Yasumoto Y, Ito M (2011). Idiopathic intervertebral disk calcification in childhood: a case report and review of literature. Childs Nerv Syst.

[9] Mori K, Imai S, Kasahara T, Nishizawa K, Mimura T, Matsusue Y (2014). Prevalence, distribution, and morphology of thoracic ossification of the posterior longitudinal ligament in Japanese: results of CT-based cross-sectional study. Spine (Phila Pa 1976).

[10] Smith ZA, Buchanan CC, Raphael D, Khoo LT (2011). Ossification of the posterior longitudinal ligament: pathogenesis, management, and current surgical approaches. A review. Neurosurg Focus.

[11] Sasaki E, Ono A, Yokoyama T, Wada K, Tanaka T, Kumagai G (2014). Prevalence and symptom of ossification of posterior longitudinal ligaments in the Japanese general population. J Orthop Sci.

[12] Boody BS, Lendner M, Vaccaro AR (2019). Ossification of the posterior longitudinal ligament in the cervical spine: a review. Int Orthop.

[13] Zhu J, Sun K, Xu X, Sun J, Kong Q, Wang S (2019). A preliminary attempt of nonintervention in the treatment of patients with intervertebral disc calcification combined with ossification of the posterior longitudinal ligament. World Neurosurg.

[14] Fu Z, Shi J, Jia L, Yuan W, Guan Z (2011). Intervertebral thoracic disc calcification associated with ossification of posterior longitudinal ligament in an eleven-year-old child. Spine (Phila Pa 1976).

[15] Li CH, Lui TH, Ngai WK (2016). Acute calcification of intervertebral disc and posterior longitudinal ligament in a 7-year-old girl: a case report. J Orthop Surg (Hong Kong).

[16] Mizukawa K, Kobayashi T, Yamada N, Hirota T (2017). Intervertebral disc calcification with ossification of the posterior longitudinal ligament. Pediatr Int.

[17] O'Dell MC, Flores M, Murray JV (2016). Pediatric idiopathic intervertebral disc calcification. Pediatr Neurol.

[18] Song XX, Yu YJ, Li XF, Liu ZD, Yu BW, Guo Z (2014). Estrogen receptor expression in lumbar intervertebral disc of the elderly: gender- and degeneration degree-related variations. Joint Bone Spine.

[19] Tomaszewski KA, Saganiak K, Gładysz T, Walocha JA (2015). The biology behind the human intervertebral disc and its endplates. Folia Morphol (Warsz).

[20] Dönmez H, Mavili E, Ikizceli T, Koç RK (2008). Pediatric intervertebral disc calcification. Diagn Interv Radiol.

[21] Nerlich AG, Schaaf R, Wälchli B, Boos N (2007). Temporo-spatial distribution of blood vessels in human lumbar intervertebral discs. Eur Spine J.

[22] Abiola R, Rubery P, Mesfin A (2016). Ossification of the posterior longitudinal ligament: etiology, diagnosis, and outcomes of nonoperative and operative management. Global Spine J.

[23] Rajasekaran S, Naresh-Babu J, Murugan S (2007). Review of postcontrast MRI studies on diffusion of human lumbar discs. J Magn Reson Imaging.

[24] El Demellawy D, Robison JG, Pollack IF, Green MD, Alper CM, Reyes-Múgica M (2013). Idiopathic intervertebral disc calcification in childhood: an atypical case of an uncommon entity for pediatric pathologists. Pediatr Dev Pathol.

[25] Swischuk LE, Jubang M, Jadhav SP (2008). Calcific discitis in children: vertebral body involvement (possible insight into etiology). Emerg Radiol.

[26] Chen J, Song D, Wang X, Shen X, Li Y, Yuan W (2011). Is ossification of posterior longitudinal ligament an enthesopathy?. Int Orthop.

[27] Sato R, Uchida K, Kobayashi S, Yayama T, Kokubo Y, Nakajima H (2007). Ossification of the posterior longitudinal ligament of the cervical spine: histopathological findings around the calcification and ossification front. J Neurosurg Spine.

